# Neutralization Activity against SARS-CoV-2 Variants after Booster Vaccination in Populations without COVID-19: A Meta-Analysis

**DOI:** 10.3390/vaccines10071101

**Published:** 2022-07-08

**Authors:** Haoyue Cheng, Zhicheng Peng, Shuting Si, Xialidan Alifu, Haibo Zhou, Peihan Chi, Yan Zhuang, Minjia Mo, Yunxian Yu

**Affiliations:** 1 Department of Public Health and Department of Anesthesiology, the Second Affiliated Hospital of Zhejiang University School of Medicine, Hangzhou 310009, China; 3150101365@zju.edu.cn (H.C.); 22018678@zju.edu.cn (Z.P.); 21818499@zju.edu.cn (S.S.); 3130100017@zju.edu.cn (X.A.); 11918158@zju.edu.cn (H.Z.); 22118872@zju.edu.cn (P.C.); yanzhuang@zju.edu.cn (Y.Z.); minjiamo@zju.edu.cn (M.M.); 2 Department of Epidemiology & Health Statistics, School of Public Health and Medicine, Zhejiang University, Hangzhou 310058, China

**Keywords:** COVID-19 vaccine, variant, booster, homologous, heterologous, neutralization

## Abstract

A number of SARS-CoV-2 variants that have evolved to have significant immune escape have emerged worldwide since the COVID-19 outbreak. The efficacy of prime vaccination is waning with the evolution of SARS-CoV-2, and the necessity of booster doses is more and more prominent. Therefore, this study aimed to compare the neutralization activity against the wild type and variants (Beta, Delta, and Omicron) in different prime–boost vaccination regimens. Electronic databases including PubMed, the Cochrane Library, Embase, medRxiv, Wanfang and CNKI were used to retrieve original studies. A total of 16 studies, 9 prime–boost vaccination regimes, and 3134 subjects were included in the meta-analysis and random effect models were used to estimate pooled neutralization titers. The neutralization activity against SARS-CoV-2 showed a significant decline with the evolution of the virus, especially in the populations primed with inactivated vaccines. For homologous immunization, only the populations boosted with mRNA vaccines consistently had a significant rise in neutralization titers (Beta: MD = 0.97; Delta: MD = 1.33; Omicron: MD = 0.74). While the heterologous immunization was more effective, the increment of neutralization titers against wild type, Beta, Delta and Omicron was 1.65 (95% CI: 1.32–1.96), 1.03 (95% CI: 0.53–1.54), 1.46 (95% CI: 1.07–1.85) and 1.15 (95% CI: 0.68–1.61), respectively. With the evolution of SARS-CoV-2, the effectiveness of prime immunization is waning. Although the administration of the booster dose could ameliorate the neutralization titers, homologous immunization regimens were gradually losing their effectiveness. Therefore, a heterologous booster dose is required, especially in populations primed with inactivated vaccines.

## 1. Introduction

Coronavirus disease 2019 (COVID-19) has spread across 200 countries with 507 million confirmed cases and over 6.2 million deaths worldwide (Johns Hopkins University Coronavirus Resource Center, https://coronavirus.jhu.edu/map.html (accessed on 21 April 2022). All viruses, including SARS-CoV-2, the virus that causes COVID-19, change over time [1]. Up to now, there have been five main variants that spread globally, i.e., Alpha (B.1.1.7), Beta (B.1.351), Gamma (P.1), Delta (B.1.617.2) and Omicron (B.1.1.529) variants [1]. Taking the Omicron variant as an example, it has quickly attracted concerns worldwide, mainly because it has over 50 mutations, of which over 30 mutations were in the region of the spike protein [2]. These mutations induced a net enhancing effect on the binding of the Omicron receptor-binding domain (RBD) to human angiotensin-converting enzyme 2 (ACE2) receptors relative to the wild type, which suggests that structural epistasis enables immune evasion while retaining efficient receptor engagement [3]. In general, evidence supports the suspicions that the variants have evolved to show significant immune escape, a higher chance of SARS-CoV-2 reinfection, and more rapid spread [4].

Although multiple effective vaccines which were developed from a variety of platforms are being promoted globally (such as CoronaVac, BNT162b2, ChAdOx1 nCoV-19, and so on), the variants still bring concerns for increasing spread and escape from the vaccines [5,6]. Therefore, WHO prompted the classification of novel SARS-CoV-2 strains as Variants of Interest (VOIs) and Variants of Concern (VOCs) [1]. One of the typical characteristics of VOCs is the decreased response to treatments and vaccines [7]. How do we deal with the challenges posed by VOCs? Some studies have emphasized the necessity of booster vaccinations [8,9,10]. However, considering the prioritization of limited vaccine resources and vaccine equity, many global health academics and governments are still awaiting data on the efficacy of third-dose boosters with different vaccines [11].

Although some studies on COVID-19 booster vaccinations have been carried out, there is still a lack of a systematic description of homologous and heterologous booster vaccination against the SARS-CoV-2 variants. Besides, many studies only estimated the vaccine effectiveness rather than immunogenicity, so they cannot accurately represent the vaccine’s efficacy against a particular SARS-CoV-2 variant [12,13]. Therefore, our study will be the first summarizing the clinical trials of COVID-19 vaccine booster shots to compare the neutralization activity against the wild type and variants (Beta, Delta, and Omicron) in different prime–boost vaccination regimens, providing a useful reference for the recommendation of booster vaccinations.

## 2. Materials and Methods

### 2.1. Search Strategy and Protocol

We followed the Cochrane Handbook for Systematic Review of Interventions [14] and the Preferred Reporting Items for Systematic Reviews and Meta-Analyses (PRISMA 2020 statement) in conducting and reporting of the meta-analysis [15]. The search was performed in PubMed, the Cochrane Library, Embase, medRxiv, Wanfang and CNKI to identify all published and pre-publication studies. Detailed search strategies for all six databases are provided in the Supplementary Materials (Tables S1–S4).

### 2.2. Eligibility Criteria

PICOS (population, intervention, comparison, outcome and study design) approach was used to define study eligibility criteria [16]:

- Population—participants received two doses of homologous COVID-19 vaccines and without history of laboratory-confirmed COVID-19;
- Intervention—booster dose of the COVID-19 vaccines;
- Comparison—before (day 0) and after (day 14/28) the booster vaccination;
- Outcomes—Neutralization activity against different types of SARS-CoV-2 variants after a booster dose was evaluated by comparing the change of neutralization titers. Long-term immunogenicity post prime vaccination and the final concentration of neutralization antibody were also analyzed.
- Study designs—Before–after studies were eligible for inclusion. Animal studies, case reports, reviews, editorials and conference abstracts were excluded.

We excluded studies that did not specify the type of COVID-19 vaccines or were not published in English or Chinese. Furthermore, the studies in which the time interval between the first immunogenicity blood sampling and the booster vaccination was more than seven days were also excluded. In case of overlapping data, the most recent and detailed study was included.

### 2.3. Data Extraction and Quality Assessment

Two authors (Haoyue Cheng and Zhicheng Peng) assessed the articles for eligibility based on the title, then the abstract, and finally the full text. Disagreements were determined by the third author (Yunxian Yu). Data extraction included: study characteristics (e.g., date of publication, author, sample size, country or study area), population demographics (e.g., sex ratio, mean age, inclusion criteria, prime–boost vaccination regimen, the interval between prime and boost) and outcomes (neutralization antibody titers against specific SARS-CoV-2 variant or wild type).

The quality of the included studies was evaluated using the Newcastle–Ottawa Scale (NOS), designed for observational and non-randomized studies [17]. The NOS contains three categories (8 subcategories), with a maximum of ten stars awardable. A score of 0–3, 4–6 and 7–10 stars was considered as a low-, moderate-, and high-quality study, respectively.

### 2.4. Outcomes

Outcomes of the meta-analysis consisted of two main parts: neutralization activity against wild type, Beta, Delta, and Omicron variants post booster vaccination, and long-term immunogenicity post prime vaccination. The final concentration of neutralization antibody was also analyzed. The neutralization titers were tested by pseudovirus neutralization test (wild type) or live virus neutralization test (SARS-CoV-2 variants) at day 0 and day 14/28 after the booster dose.

### 2.5. Data Synthesis and Statistical Analysis

Data analysis was conducted as recommended in the Cochrane Handbook for Systematic Reviews of Interventions [14]. Neutralization titers were log-transformed (Log10) and converted to the arithmetic mean before the analysis. Since the neutralization of Omicron was undetectable post prime immunization, we set the mean of neutralization titers in each study as half of the detection limit and the standard deviation as 0.0001. Forest plots were constructed showing the summary and 95% confidence interval (CI) estimated in the meta-analysis. We assessed heterogeneity among studies using the *χ*^2^-based Q test and *I*^2^ statistical parameter. Therefore, simple random effect models were used due to all *I*^2^ > 50% and *p* < 0.01. We used inverse variances that incorporated an estimate of the between-study variance to calculate the weights for the model. Furthermore, all pooled outcomes were stratified across groups of the vaccination regimens.

Two independent-sample *t* test was used to compare the differences in immunogenicity between different vaccination regimens. All the statistical analyses were conducted using R statistical software VERSION 4.0.0 (The R Project for Statistical Computing).

## 3. Results

### 3.1. Characteristics of the Studies

**Figure 1:**
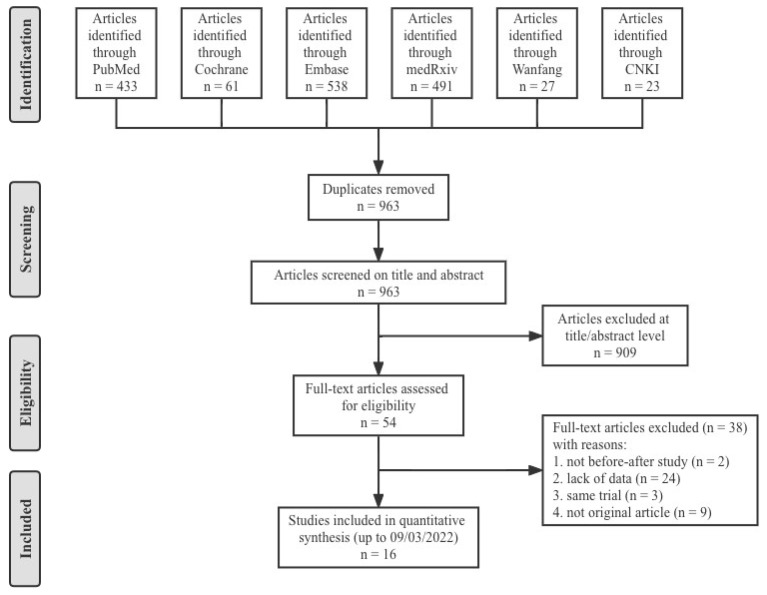
Flow diagram showing the progress through the stages of meta-analysis.

A total of 1573 articles from PubMed (433), the Cochrane Library (61), Embase (538), medRxiv (491), Wanfang (27) and CNKI (23) were included initially. After duplicates removal and screening of 963 titles and abstracts, 54 potentially eligible studies were selected for full review. Finally, we included 16 studies [2,18,19,20,21,22,23,24,25,26,27,28,29,30,31,32] that provided data on nine COVID-19 prime–boost regimens (including homologous immunization and heterologous immunization) (Figure 1). All included articles were before–after studies and seven COVID-19 vaccines were involved: two inactivated vaccines (CoronaVac and BBIBP-CorV), two mRNA vaccines (mRNA-1273 and BNT162b2), two viral vector vaccines (ChAdOx1 nCoV-19 and Ad26.COV2.S) and one recombinant protein vaccine (ZF2001). The specific nine groups of prime–boost regimens were as follows (prime–boost): inactivated–inactivated, mRNA–mRNA, viral vector–viral vector, inactivated–mRNA, inactivated–viral vector, inactivated–recombinant protein, mRNA–viral vector, viral vector–inactivated and viral vector–mRNA. General characteristics of the included studies are presented in Table 1. The study populations (a total of 3134 subjects) were all general populations without history of laboratory-confirmed COVID-19. Therefore, the antibody responses were totally induced by the vaccines. The quality of the studies, evaluated by the NOS, are also provided in Table 1. Fifteen studies had a score of 7 to 9, indicating good quality and one study had a score of 6, indicating moderate quality.

### 3.2. Long-Term Neutralization Activity against SARS-CoV-2 Variants Post Prime Immunization

**Figure 2:**
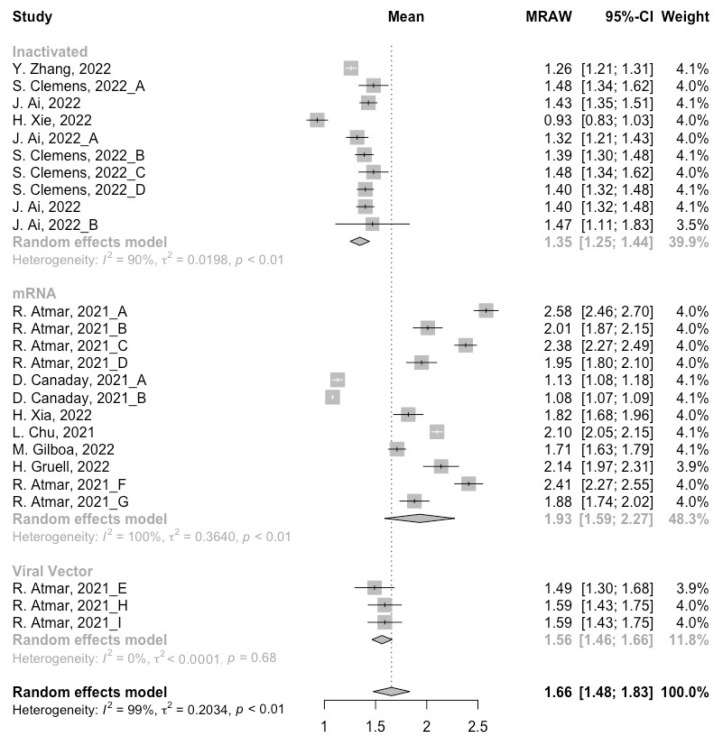
Forest plot of the pooled log-transformed neutralization titers against the wild type before booster vaccination [2,18,19,20,21,22,23,24,25,26,27,28,29,30,31,32].

**Figure 3:**
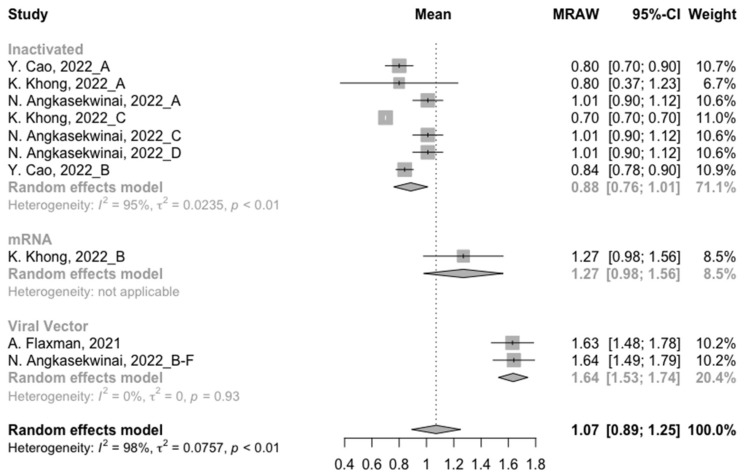
Forest plot of the pooled log-transformed neutralization titers against the Beta variant before booster vaccination [2,18,19,20,21,22,23,24,25,26,27,28,29,30,31,32].

**Figure 4:**
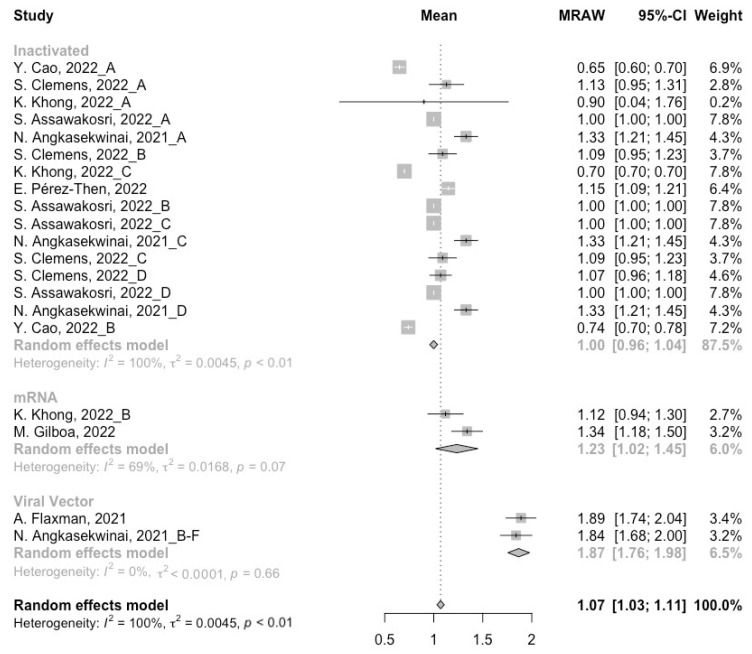
Forest plot of the pooled log-transformed neutralization titers against the Delta variant before booster vaccination [2,18,19,20,21,22,23,24,25,26,27,28,29,30,31,32].

At least 3 months after the prime vaccination, the levels of neutralization antibody titers against wild type, Beta and Delta variants were 1.66 (95% CI: 1.48–1.83), 1.07 (95% CI: 0.89–1.25) and 1.07 (95% CI: 1.03–1.11), respectively (Figure 2, Figure 3 and Figure 4). For immunity against the Omicron variant, neutralization of Omicron was undetectable post prime immunization, which might be due to the administration of inactivated vaccines in almost all studies. It is worth noting that the neutralization activity against SARS-CoV-2 in general populations showed a significant decline with the evolution of the virus. We further divided the COVID-19 vaccines into three categories according to their types to assess the difference between their long-term immunogenicity. For the wild type, populations primed with the mRNA vaccines showed the highest level of neutralization antibody titers (MRAW = 1.93, 95% CI: 1.59–2.27) (Figure 2). However, for the Beta and Delta variants, the long-term immunogenicity of the viral vector vaccines was the best (Beta: MRAW = 1.64, 95% CI: 1.53–1.74; Delta: MRAW = 1.87, 95% CI: 1.76–1.98) (Figure 3 and Figure 4).

### 3.3. Neutralization Activity against SARS-CoV-2 Variants Post Homologous Boosters

**Figure 5:**
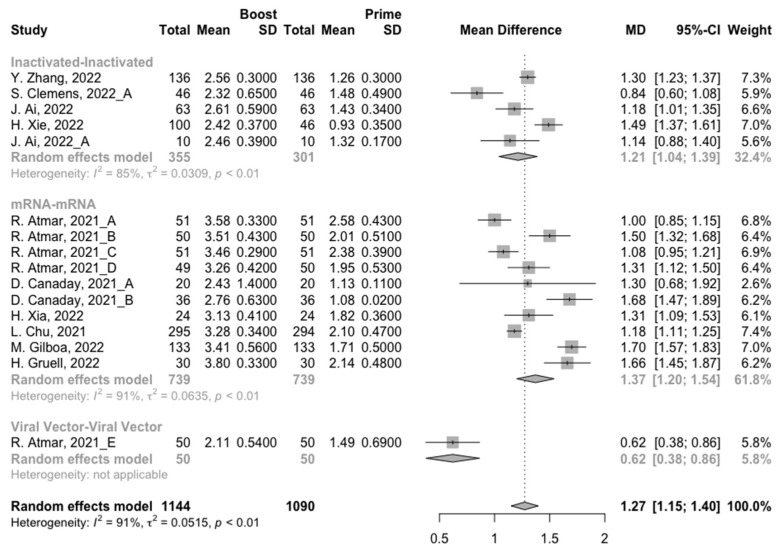
Forest plot of the pooled log-transformed neutralization titers against the wild type before and after homologous booster vaccination [2,18,19,20,21,22,23,24,25,26,27,28,29,30,31,32].

**Figure 6:**
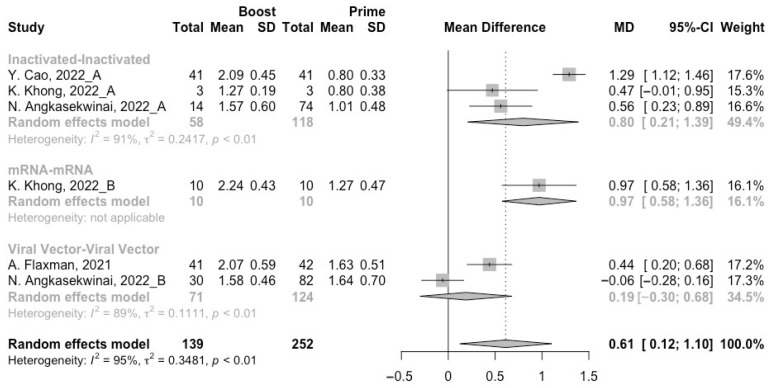
Forest plot of the pooled log-transformed neutralization titers against the Beta variant before and after homologous booster vaccination [2,18,19,20,21,22,23,24,25,26,27,28,29,30,31,32].

**Figure 7:**
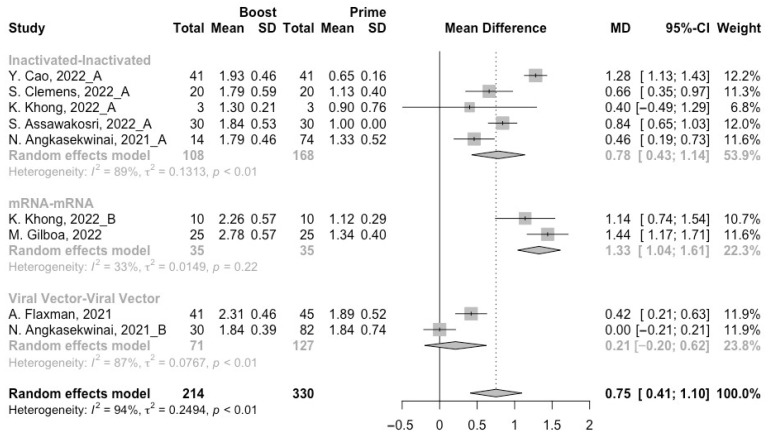
Forest plot of the pooled log-transformed neutralization titers against the Delta variant before and after homologous booster vaccination [2,18,19,20,21,22,23,24,25,26,27,28,29,30,31,32].

**Figure 8:**
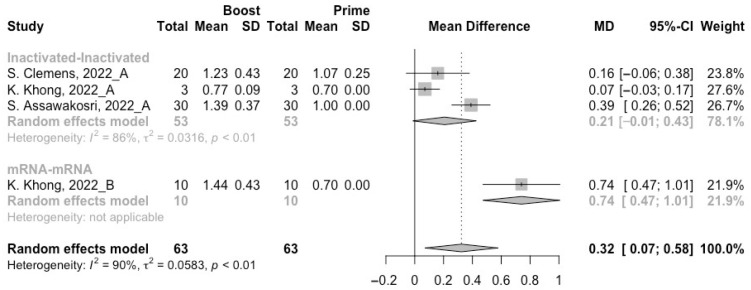
Forest plot of the pooled log-transformed neutralization titers against the Omicron variant before and after homologous booster vaccination [2,18,19,20,21,22,23,24,25,26,27,28,29,30,31,32].

To assess neutralization activity in the homologous vaccination regimens, we compared neutralization titers against wild type, Beta, Delta and Omicron variants before and after a booster dose. The increment of neutralization titers against SARS-CoV-2 variants (wild type, Beta, Delta and Omicron variants) were 1.27 (95% CI: 1.15–1.40), 0.61 (95% CI: 0.12–1.10), 0.75 (95% CI: 0.41–1.10), and 0.32 (95% CI: 0.07–0.58) at 14/28 days after receiving a booster dose, respectively (Figure 5, Figure 6, Figure 7 and Figure 8). However, with the evolution of SARS-CoV-2, only the populations boosted with homologous mRNA vaccines consistently had a significant rise in neutralization titers. For the populations boosted with homologous inactivated vaccines, a booster dose could not protect them from the Omicron variant (MD = 0.21, 95% CI: −0.01–0.43) (Figure 8). We were surprised to find that the administration of the third dose of viral vector vaccine did not show significant increment in neutralization titers against the Beta (MD = 0.19, 95% CI: −0.30–0.68) and Delta (MD = 0.21, 95% CI: −0.20–0.62) variants (Figure 6 and Figure 7).

In general, regardless of the specific type of SARS-CoV-2 variants, the final neutralization titers of homologous mRNA prime–boost vaccination were significantly higher than the other two types of vaccines (*p* < 0.05). Among the populations primed with mRNA vaccines, the levels of neutralization antibody titers against wild type, Beta, Delta and Omicron variants at 14/28 days post booster vaccination increased to 3.33 (95% CI: 3.20–3.47), 2.24 (95% CI: 1.97–2.51), 2.54 (95% CI: 2.03–3.05) and 1.44 (95% CI: 1.17–1.71), respectively (Figures S1–S4).

### 3.4. Neutralization Activity against SARS-CoV-2 Variants Post Heterologous Boosters

**Figure 9:**
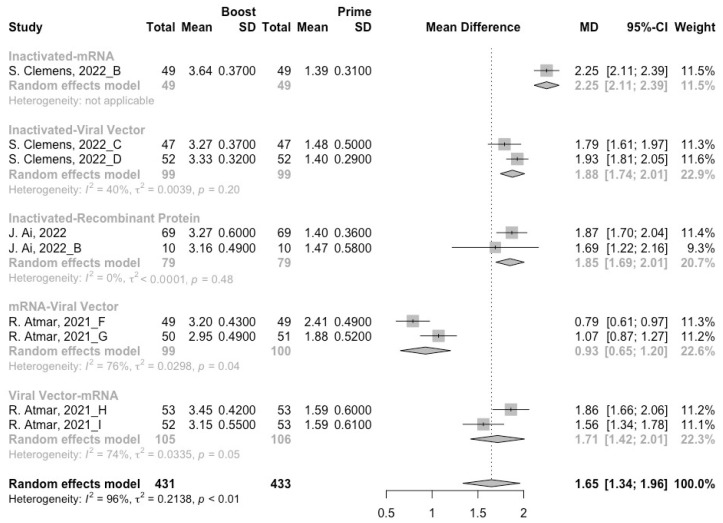
Forest plot of the pooled log-transformed neutralization titers against the wild type before and after heterologous booster vaccination [2,18,19,20,21,22,23,24,25,26,27,28,29,30,31,32].

**Figure 10:**
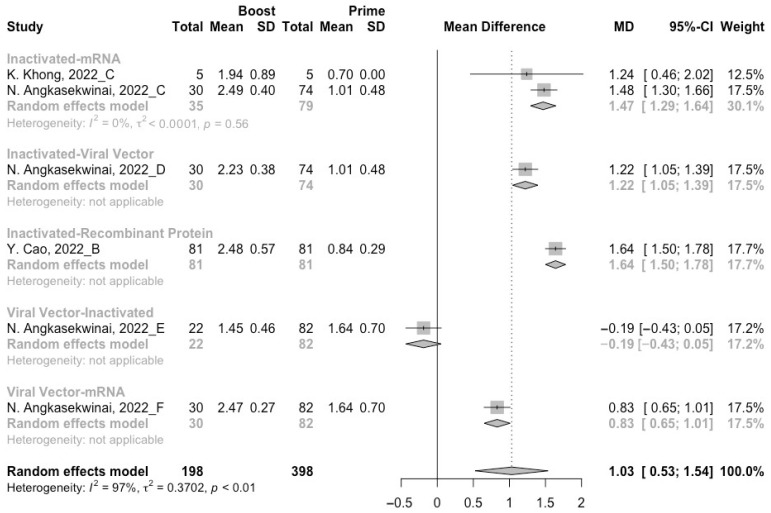
Forest plot of the pooled log-transformed neutralization titers against the Beta variant before and after heterologous booster vaccination [2,18,19,20,21,22,23,24,25,26,27,28,29,30,31,32].

**Figure 11:**
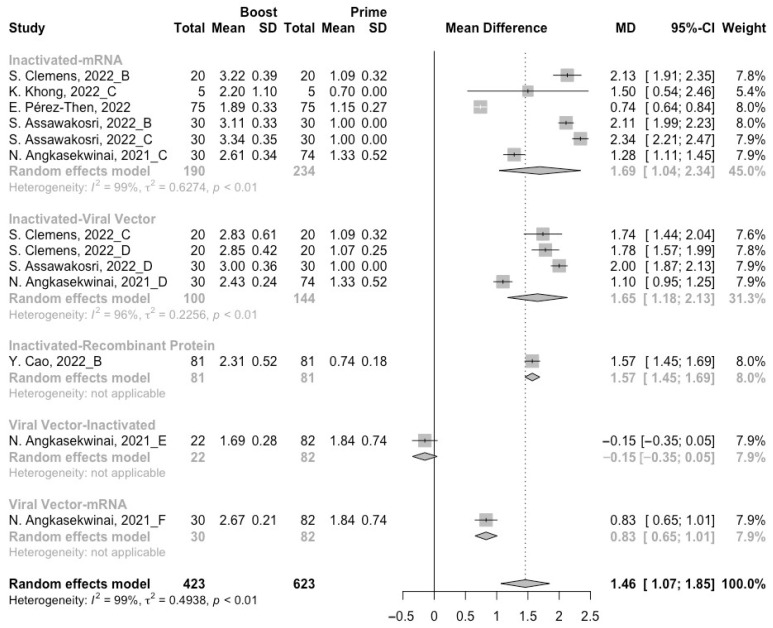
Forest plot of the pooled log-transformed neutralization titers against the Delta variant before and after heterologous booster vaccination [2,18,19,20,21,22,23,24,25,26,27,28,29,30,31,32].

**Figure 12:**
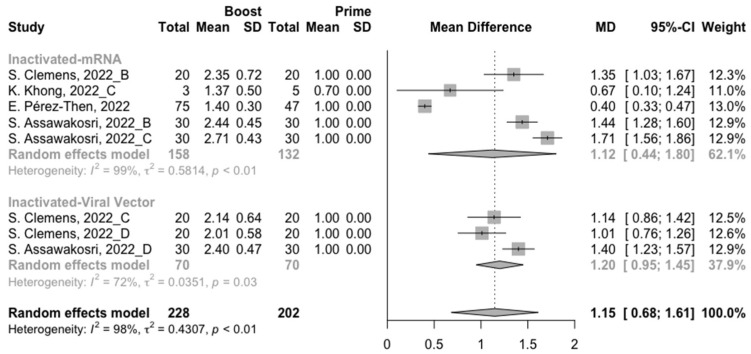
Forest plot of the pooled log-transformed neutralization titers against the Omicron variant before and after heterologous booster vaccination [2,18,19,20,21,22,23,24,25,26,27,28,29,30,31,32].

A total of six groups of heterologous vaccination regimens (inactivated–mRNA, inactivated–viral vector, inactivated–recombinant protein, mRNA–viral vector, viral vector–inactivated, and viral vector–mRNA) were included in this meta-analysis, and most populations were vaccinated with the inactivated–mRNA regimen. For all types of vaccination regimens (except the viral vector–inactivated group), populations developed neutralization activity against wild type, Beta, Delta and Omicron variants after the administration of a booster dose. The pooled increment of neutralization titers was 1.65 (95% CI: 1.32–1.96), 1.03 (95% CI: 0.53–1.54), 1.46 (95% CI: 1.07–1.85) and 1.15 (95% CI: 0.68–1.61), respectively (Figure 9, Figure 10, Figure 11 and Figure 12). After 14/28 days of a booster dose, the neutralization titers of wild type, Beta, Delta, and Omicron variants were 3.28 (95% CI: 3.14–3.41), 2.21 (95% CI: 1.92–2.49), 2.64 (95% CI: 2.35–2.93) and 2.11 (95% CI: 1.65–2.58), respectively (Figures S5–S8). Therefore, all heterologous vaccination regimens were superior to the homologous regimens (*p* < 0.01).

We further compared the difference in neutralization activity between different heterologous vaccination regimens. No matter what type of SARS-CoV-2 variant, the populations boosted with heterologous boosters after inactivated vaccines had the highest increment in neutralization titers than those primed with other types of vaccines (Figure 9, Figure 10, Figure 11 and Figure 12). However, it could not be ignored that even in the populations that received heterologous immunization regimens, the improvement in neutralization activity also showed a significant decline with the evolution of SARS-CoV-2.

## 4. Discussion

In this meta-analysis of before–after studies, we found that the neutralization activity against SARS-CoV-2 in general populations showed a significant decline with the evolution of the virus. This was especially the case for immunity against the Omicron variant, as neutralization of Omicron was undetectable post prime immunization. Among the homologous regimens, it is worth noting that only the mRNA vaccines could consistently ameliorate the neutralization activity with the evolution of the virus. Regardless of the type of SARS-CoV-2 variant, the immunogenicity of heterologous vaccination regimens was superior to the homologous regimens, especially in the populations primed with inactivated vaccines.

The emergence of novel variants of SARS-CoV-2 highlights one of the primary challenges facing the COVID-19 pandemic [33]. Using previous data on the effectiveness of vaccines against the earlier variants, Gradner et al. [34] developed computer models that indicated that after two doses of mRNA vaccines, the efficacy against symptomatic infection from Delta was 87%, while the efficacy against the Omicron variant was only 30%. More and more studies have also confirmed that current and new variants will impact the spread of SARS-CoV-2 and the efficacy of vaccines [5,35]. The results of this meta-analysis are consistent with the above studies, especially the alarming decline in the neutralization activity of the inactivated vaccines with the evolution of the virus. However, why does the effectiveness of the vaccines decline with the evolution of the virus? Selective pressure, which is one of the main driving forces of viral mutation, promotes a mutation that permits SARS-CoV-2 to escape from immune responses. The spike protein, the most critical surface protein of SARS-CoV-2, plays a vital role in the binding of the virus to, and its fusion with, the host cell membrane receptor [36]. Given its crucial role in SARS-CoV-2 natural infection and adaptive immunity, the spike protein is an important target site for neutralization antibodies and a key target for current vaccine design [5,37]. Therefore, mutations in the spike protein have the potential to affect the binding of antibodies to host receptors, resulting in changes in the infectivity and transmission efficiency of the virus, and its immune escape from neutralization after vaccination [37]. As of now, all VOCs contain mutations in the spike protein. Taking the mutations on the RBD of the spike protein as an example, N501Y in the Alpha, Beta, and Gamma variants increased transmissibility [38], and E484K could affect the effectiveness of the vaccines [39]. Therefore, the number of SARS-CoV-2 mutations might be a critical reason behind the gradual decline in the effectiveness of the vaccines. In general, there is no doubt that the immunogenicity of the COVID-19 vaccine is declining with the evolution of the virus, which has been confirmed by both theory and reality.

Vaccine effectiveness is determined by neutralization antibodies, which prevent SARS-CoV-2 from getting into the cells, and T-cells, which attack infected cells and help with antibody production [40]. The meta-analysis confirmed the decline of neutralization activity against SARS-CoV-2 variants after prime vaccination. However, a booster dose is a “trigger” that can stimulate B cells to secrete more neutralization antibodies to prevent the invasion of SARS-CoV-2 [41]. The improvement in immunogenicity after the administration of the booster dose is also effective against the variants because they still bind the host cell membrane receptor via the spike protein. The result of our meta-analysis confirmed that a booster dose also exhibited potent neutralization titers against the variants, and it indicated that the immunogenicity of heterologous immunization was much better. The mechanism for this difference is that using dissimilar platforms can induce protection from different pathways [42]. The theoretical advantage of inactivated vaccines is that they contain additional viral proteins, such as nucleoprotein, which can potentially extend the protection beyond anti-spike protein responses [22]. As an emerging technology, mRNA vaccines are based on the theory that mRNA is an intermediate messenger that can be easily delivered into host cells and translated into antigens that will trigger a protective antigen-specific immune response [33,43]. Therefore, mRNA vaccines have dual mechanisms of humoral immunity and T-cell immunity, and have strong immunogenicity. As for viral vector vaccines, they also induce humoral immunity and cell-mediated immunity [33]. Moreover, viral vector vaccines can elicit long-lasting immune responses immediately after only one dose of vaccine [44].

However, it is worth noting that our meta-analysis found that the administration of a booster dose of viral vector vaccine did not show significant increment in neutralization titers against the variants. A possible explanation could be the immune response to the adenovirus vector backbone (antivector immunity) [45]. Specifically, some studies noticed that pre-existing anti-adenovirus immunity of the participants could affect the vaccine’s safety and immunogenicity [46,47]. Taking Ad5-nCoV as an example, Zhu et al. [46] found that the participants with higher baseline neutralizing antibodies to Ad5 were more tolerant of a booster dose. Furthermore, some experts have indicated that T-cells respond to the whole of the spike protein, so they are less likely to be bothered by a few mutations [40]. Therefore, mRNA and viral vector vaccines may still maintain high immunogenicity of T-cell responses. Unfortunately, since the original studies did not provide enough data on T cell responses, this meta-analysis could not verify the mechanism. In addition to the type of COVID-19 booster vaccine, Chiu et al. [48] found that the order of prime–boost also mattered, and the results of our study showed that the immunogenicity of the inactivated–viral vector vaccination regimen was better than that of the viral vector–inactivated regimen. Therefore, further studies are required to find out the best order of vaccinations.

There are several limitations in our meta-analysis. First, the neutralization titers were tested by different methods (wild type: pseudovirus neutralization test; variants: live virus neutralization test). Therefore, the comparability between them was weak. However, Sholukh et al. [49] confirmed that the high concordance between the outcomes of live and pseudotyped neutralization tests supported valid cross-study comparison using these platforms. Second, most of the original studies were limited to several countries, such as China, USA and Thailand. Due to government policy, individuals in different countries had to accept a certain type of vaccine. Governments in Europe and North America were more inclined to promote the mRNA vaccines, while governments in Asia preferred the inactivated vaccines. Therefore, the representativeness of the meta-analysis might be affected by race, vaccination strategy, and so on. Third, the interval between prime and boost influenced the immunogenicity of the vaccines [48]. This is one of the possible reasons for the high heterogeneity in the meta-analysis. Fourth, when the studies were stratified by the type of vaccines, the number of studies in each group was inadequate. Furthermore, the sample sizes in some studies were small. Therefore, achieving adequate statistical power might be difficult, and a cautious approach in interpreting the results is warranted.

In general, our study pointed out the importance of heterologous booster vaccinations against SARS-CoV-2 variants. However, as the booster doses are not properly enhancing natural immunity for longer periods, at least for the Omicron case, updating the vaccines against new emergent variants is critical. On 25 January 2022, Pfizer-BioNTech announced the initiation of a clinical study to evaluate the safety, tolerability and immunogenicity of an Omicron-based vaccine [50]. Additionally, Sinovac also developed an Omicron-based inactivated vaccine [51]. Therefore, it is necessary to continue to pay attention to the immunogenicity and safety of the new vaccines. Furthermore, some companies have also developed monoclonal antibodies (mAbs). Compared with polyclonal antibodies induced by the vaccines, some mAbs have shown remarkable potency and resistance to many VoCs [52], and mAbs could play a protective effect immediately after injection. If the high costs of mAbs are solved, it may also become one of the important means of preventing COVID-19 [53].

## 5. Conclusions

The meta-analysis summarized the neutralization activity against the wild type and variants (Beta, Delta, and Omicron) of SARS-CoV-2 in different prime–boost vaccination regimens. With the evolution of SARS-CoV-2, the neutralization activity against specific variants has declined in general populations. Although the administration of the booster dose can ameliorate the neutralization titers, homologous immunization regimens have gradually been losing their effectiveness. Considering the global outbreak of the Omicron variant and more future mutations of SARS-CoV-2, a heterologous booster dose is required, especially in populations primed with inactivated vaccines.

## Figures and Tables

**Table 1:** Characteristics of the original studies included in the meta-analysis.

Study and Year	Country	Number of Groups	Participants (N)	Characteristics of the Participants ^1^	Age (Mean/Median)	Male (%)	COVID-19 Vaccines (Prime/Boost) ^2^	Interval of Boost	SARS-CoV-2 Variants	NOS Score
Atmar et al., 2021 [2]	USA	9	51; 50; 51; 50; 50; 49; 51; 53; 53	Healthy adults	53.1; 54.8; 54.3; 50.4; 50.1; 49.9; 50.3; 56.8; 47.7	37.3; 42; 49; 54; 54; 67.3; 54.9; 50.9; 45.3	mRNA-1273/mRNA-1273; BNT/mRNA-1273; mRNA-1273/BNT; BNT/BNT; Ad26/Ad26; mRNA-1273/Ad26; BNT/Ad26; Ad26/mRNA-1273; Ad26/BNT	at least 12 weeks	wild type, Beta, Delta	8
Flaxman et al., 2021 [18]	UK	1	75	Healthy adults	37	60	ChAd/ChAd	20–38 weeks	wild type, Alpha, Beta, Delta	6
Cao et al., 2022 [19]	China	2	41; 81	Healthy adults	38.1; 40.7	24.4; 30.9	CoronaVac/CoronaVac; CoronaVac/ZF2001	4–8 months	wild type, Beta, Delta	9
Ai et al., 2022 [20]	China	1	69	Healthy adults	28	43.7	BBIBP/ZF2001	4–8 months	wild type, Alpha, Beta, Delta	9
Clemens et al., 2022 [21]	Brazil	4	281; 333; 295; 296	Healthy adults	60	39.5	CoronaVac/CoronaVac; CoronaVac/BNT; CoronaVac/Ad26; CoronaVac/ChAd	6 months	wild type, Delta, Omicron	9
Ai et al., 2022 [22]	China	1	63	Healthy adults	28	42.9	BBIBP/BBIBP	4–8 months	wild type, Alpha, Beta, Delta	9
Khong et al., 2022 [23]	China	3	3; 10; 5	Healthy adults	58; 53; 58.5	55.6; 46.7; 37.5	CoronaVac/CoronaVac; BNT/BNT; CoronaVac/BNT	at least 6 months	wild type, Beta, Delta, Omicron	6
Xie et al., 2022 [24]	China	1	46	Healthy adults aged 18–59 years	NA	NA	CoronaVac/CoronaVac	at least 12 months	wild type, Alpha, Beta, Delta	8
Xia et al., 2022 [25]	USA	1	24	Healthy adults	52.9	37.5	BNT/BNT	NA	wild type, Omicron	8
Chu et al., 2021 [26]	USA	1	295	Healthy adults	52	33.7	mRNA-1273/mRNA-1273	7.2 ± 0.6 months	wild type, Delta	9
Gilboa et al., 2022 [27]	Israel	1	159	Healthy adults aged 60 years and older	66	35	BNT/BNT	NA	wild type, Delta	8
Gruell et al., 2022 [28]	Germany	1	30	Healthy adults	49	43	BNT/BNT	26–41 weeks	wild type, Omicron	7
Ai et al., 2022 [29]	China	2	10; 10	Healthy adults	27; 24.5	60; 60	BBIBP/BBIBP; BBIBP/ZF2001	4–8 months	wild type, Beta, Delta, Omicron	8
Pérez-Then et al., 2022 [30]	The Dominican Republic	1	75	Healthy adults	40.4	30	CoronaVac/BNT	109.5 ± 34.9 days	wild type, Delta, Omicron	8
Assawakosri et al., 2022 [31]	Thailand	4	57; 54; 58; 55	Healthy adults	41.9; 41.6; 37; 44.1	40.4; 59.3; 47.8; 43.6	CoronaVac/BBIBP; CoronaVac/BNT; CoronaVac/mRNA-1273; CoronaVac/ChAd	5–7 months	wild type, Delta, Omicron	9
Angkasekwinai et al., 2021 [32]	Thailand	6	14; 50; 50; 65; 23; 49	Healthy adults	31; 45.5; 32; 36.6; 51; 34	14.3; 6; 20; 21.5; 8.7; 26	CoronaVac/BBIBP; ChAd/ChAd; CoronaVac/BNT; CoronaVac/ChAd; ChAd/BBIBP; ChAd/BNT	8–12 weeks	wild type, Beta, Delta	8

^1^ All studies recruited participants without history of laboratory-confirmed COVID-19. ^2^ BNT: BNT162b2; Ad26: Ad26.COV2.S; Ad5: Ad5-nCoV; ChAd: ChAdOx1 nCoV-19; BBIBP: BBIBP-CorV.

## Data Availability

The data presented in this study are available in the article.

## Supplementary Materials

The following supporting information can be downloaded at: https://www.mdpi.com/article/10.3390/vaccines10071101/s1, Table S1: Literature search terms used for PubMed. The final search strategy applied to conduct this meta-analysis is reported at step #15, Table S2: Literature search terms used for the Cochrane Library. The final search strategy applied to conduct this meta-analysis is reported at step #20, Table S3: Literature search terms used for Embase. The final search strategy applied to conduct this meta-analysis is reported at step #18, Table S4: Literature search terms used for Medrxiv, Wanfang and CNKI; Figure S1: Forest plot of the pooled log-transformed neutralization titers against the wild type after homologous booster vaccination, Figure S2: Forest plot of the pooled log-transformed neutralization titers against the Beta variant after homologous booster vaccination, Figure S3: Forest plot of the pooled log-transformed neutralization titers against the Delta variant after homologous booster vaccination, Figure S4: Forest plot of the pooled log-transformed neutralization titers against the Omicron variant after homologous booster vaccination, Figure S5: Forest plot of the pooled log-transformed neutralization titers against the wild type after heterogenous booster vaccination, Figure S6: Forest plot of the pooled log-transformed neutralization titers against the Beta variant after heterogenous booster vaccination, Figure S7: Forest plot of the pooled log-transformed neutralization titers against the Delta variant after heterogenous booster vaccination, Figure S8: Forest plot of the pooled log-transformed neutralization titers against the Omicron variant after heterogenous booster vaccination.
