# Supplementary material for: Neutralization Activity against SARS-CoV-2 Variants after Booster Vaccination in Populations without COVID-19: A Meta-Analysis
Source: Vaccines (Basel). 2022 Jul 8;10(7):1101. doi: 10.3390/vaccines10071101 (PMC9322873; doi:10.3390/vaccines10071101)
Supplement: Supplementary file 1 [file vaccines-10-01101-s001.zip › vaccines-1723411-supplementary.pdf]

Supplementary Materials

Table S1. Literature search terms used for PubMed. The final search strategy applied to conduct this meta-analysis is reported at step #15.

#	**Search strategy**
1	COVID19[tiab] OR COVID-19[tiab] OR COVID2019[tiab] OR COVID-2019[tiab]
2	SARS-CoV-2[tiab] OR sarscov2[tiab]
3	#1 OR #2
4	vaccin*[tiab] OR immun*[tiab]
5	#3 AND #4 (46840)
6	"COVID-19 Vaccines"[Mesh]
7	#5 OR #6 (48166)
8	boost*[tiab] OR third[tiab]
9	#7 AND #8 (2759)
10	variant*[tiab] OR mutation*[tiab]
11	wild type[tiab] OR alpha[tiab] OR beta[tiab] OR gamma[tiab] OR delta[tiab] OR omicron[tiab]
12	#10 OR #11
13	#9 AND #12 (563)
14	Humans[Mesh] OR human*[tiab] OR volunteer*[tiab] OR participant*[tiab] OR subject*[tiab] OR people[tiab]
15	#13 AND #14 (433)

Table S2. Literature search terms used for the Cochrane Library. The final search strategy applied to conduct this meta-analysis is reported at step #20.

#	**Search strategy**
1	MeSH descriptor: [COVID-19] explode all trees
2	(COVID19):ti,ab,kw OR (COVID 19):ti,ab,kw OR (COVID 2019):ti,ab,kw
3	MeSH descriptor: [SARS-CoV-2] explode all trees
4	(SARS CoV 2):ti,ab,kw OR (sarscov2):ti,ab,kw
5	#1 OR #2 OR #3 OR #4
6	MeSH descriptor: [Immunogenicity, Vaccine] explode all trees
7	(vaccin*):ti,ab,kw OR (immun*):ti,ab,kw OR (inocul*):ti,ab,kw
8	#6 OR #7
9	#5 AND #8 (2542)
10	MeSH descriptor: [COVID-19 Vaccines] explode all trees
11	#9 OR #10 (2542)
12	(boost*):ti,ab,kw OR (third):ti,ab,kw
13	#11 AND #12 (291)
14	(wild type):ti,ab,kw OR (alpha):ti,ab,kw OR (beta):ti,ab,kw OR (gamma):ti,ab,kw OR (delta):ti,ab,kw OR (omicron):ti,ab,kw
15	(variant*):ti,ab,kw OR (mutation*):ti,ab,kw
16	#14 OR #15
17	#13 AND #16 (63)
18	(human*):ti,ab,kw OR (volunteer*):ti,ab,kw OR (participant*):ti,ab,kw OR (subject*):ti,ab,kw OR (people*):ti,ab,kw
19	#17 AND #18 (62)
20	trial: 61

Table S3. Literature search terms used for Embase. The final search strategy applied to conduct this meta-analysis is reported at step #18.

#	**Search strategy**
1	'coronavirus disease 2019'/exp OR 'coronavirus disease 2019' OR 'covid-19' OR covid19 OR sarscov2 OR 'covid-2019' OR covid2019
2	'severe acute respiratory syndrome coronavirus 2'/exp OR 'ncov' OR 'sars-cov-2'
3	#1 OR #2
4	'vaccine'/exp OR vaccin*:ti,ab,kw
5	'immunization'/exp OR immun*:ti,ab,kw
6	inocul*:ti,ab,kw
7	#4 OR #5 OR #6
8	#3 AND #7 (53201)
9	'sars-cov-2 vaccine'/exp OR 'sars-cov-2 vaccine' OR "covid19 vaccin*" OR "covid 19 vaccin*"
10	#8 OR #9 (53309)
11	boost*:ti,ab,kw OR third:ti,ab,kw
12	#10 AND #11 (2931)
13	'wild type':ti,ab,kw OR alpha:ti,ab,kw OR beta:ti,ab,kw OR gamma:ti,ab,kw OR delta:ti,ab,kw OR omicron:ti,ab,kw
14	variant*:ti,ab,kw OR mutation*:ti,ab,kw
15	#13 OR #14
16	#12 AND #15 (599)
17	'human'/exp OR human:ti,ab,kw OR volunteer*:ti,ab,kw OR participant*:ti,ab,kw OR subject*:ti,ab,kw OR people:ti,ab,kw
18	#16 AND #17 (538)

Table S4. Literature search terms used for Medrxiv, Wanfang and CNKI.

**Database**	**Search strategy**
Medrxiv	COVID-19 AND vaccin* AND (boost* OR third) AND (variant*) (491)
Wanfang	(Theme:(COVID-19) or Theme:(SARS-CoV-2)) and Theme:(Vaccine*) and (Theme:(boost*) or Theme:(boost immunity) or Theme:(third dose)) and Theme:(variant*) (27)
CNKI	TKA=('COVID-19'+'SARS-CoV-2') and TKA=('vaccine*') and TKA=('boost*'+'third dose') and TKA=('variant*') (23)

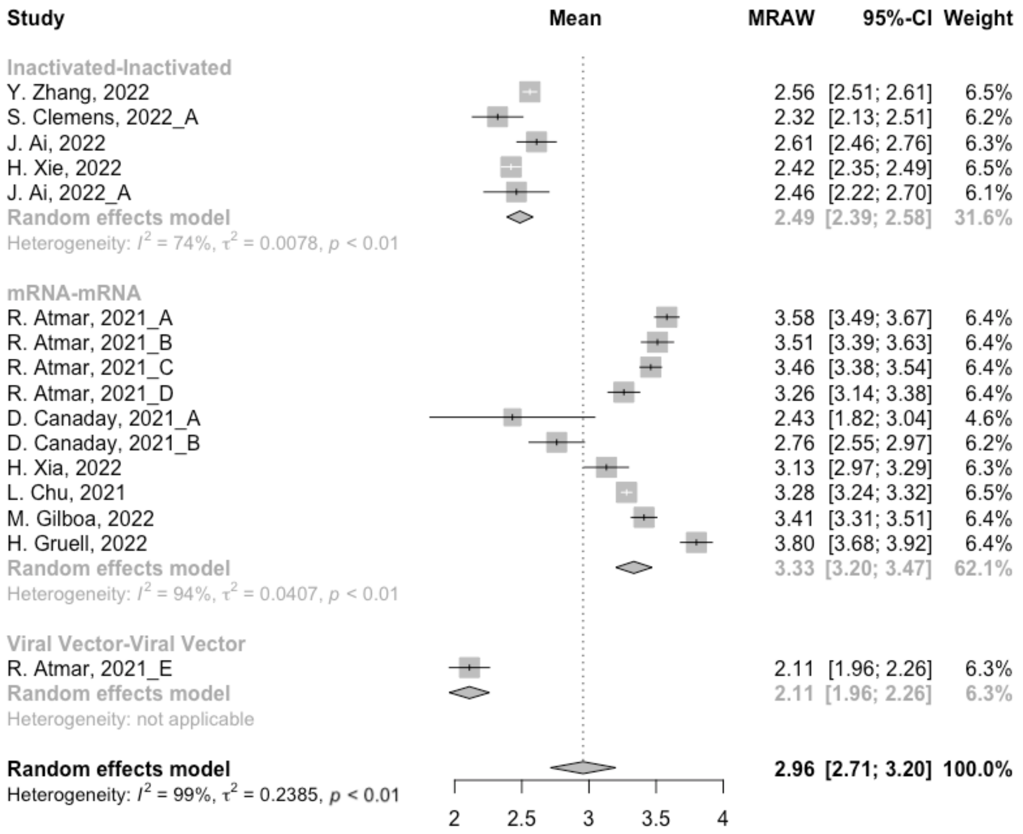

Figure S1. Forest plot of the pooled log-transformed neutralization titers against the wild type after homologous booster vaccination.

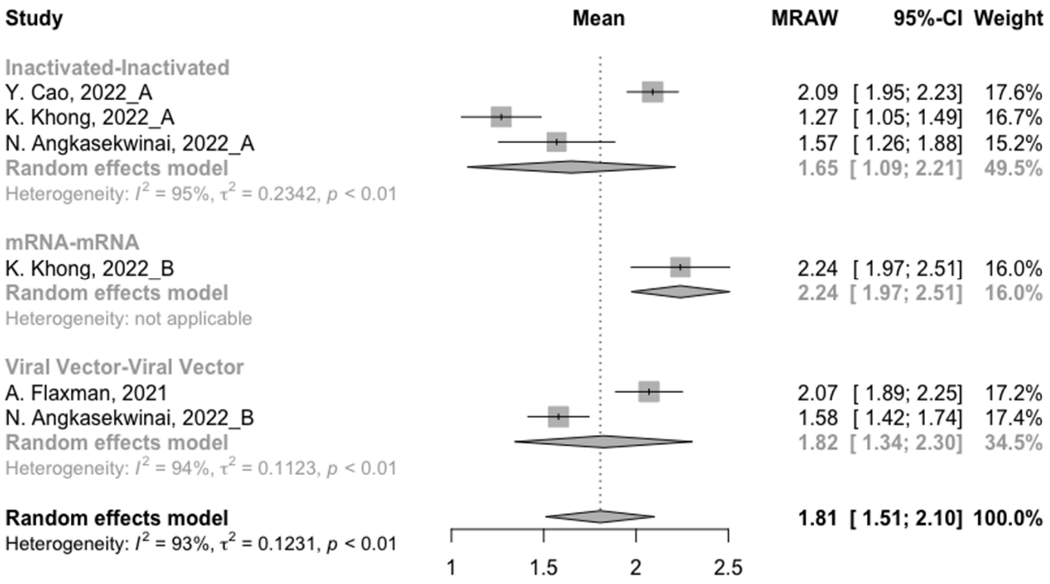

Figure S2. Forest plot of the pooled log-transformed neutralization titers against the Beta variant after homologous booster vaccination.

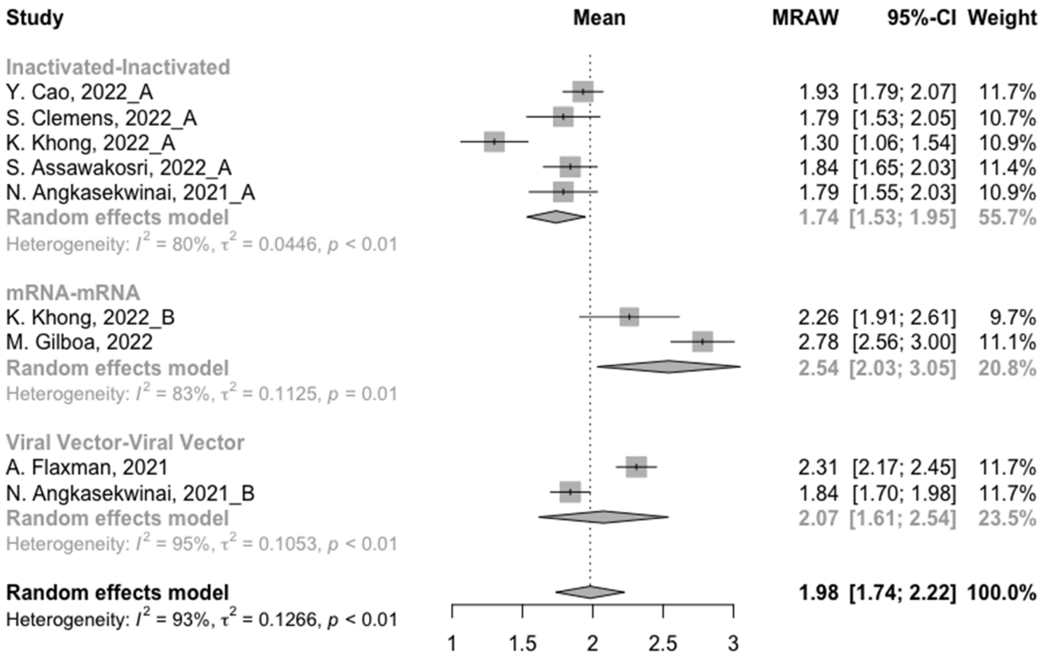

Figure S3. Forest plot of the pooled log-transformed neutralization titers against the Delta variant after homologous booster vaccination.

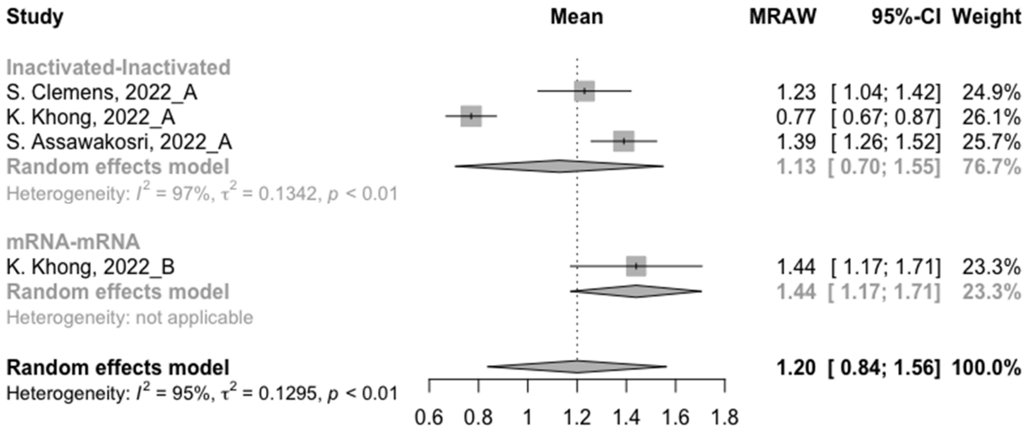

Figure S4. Forest plot of the pooled log-transformed neutralization titers against the Omicron variant after homologous booster vaccination.

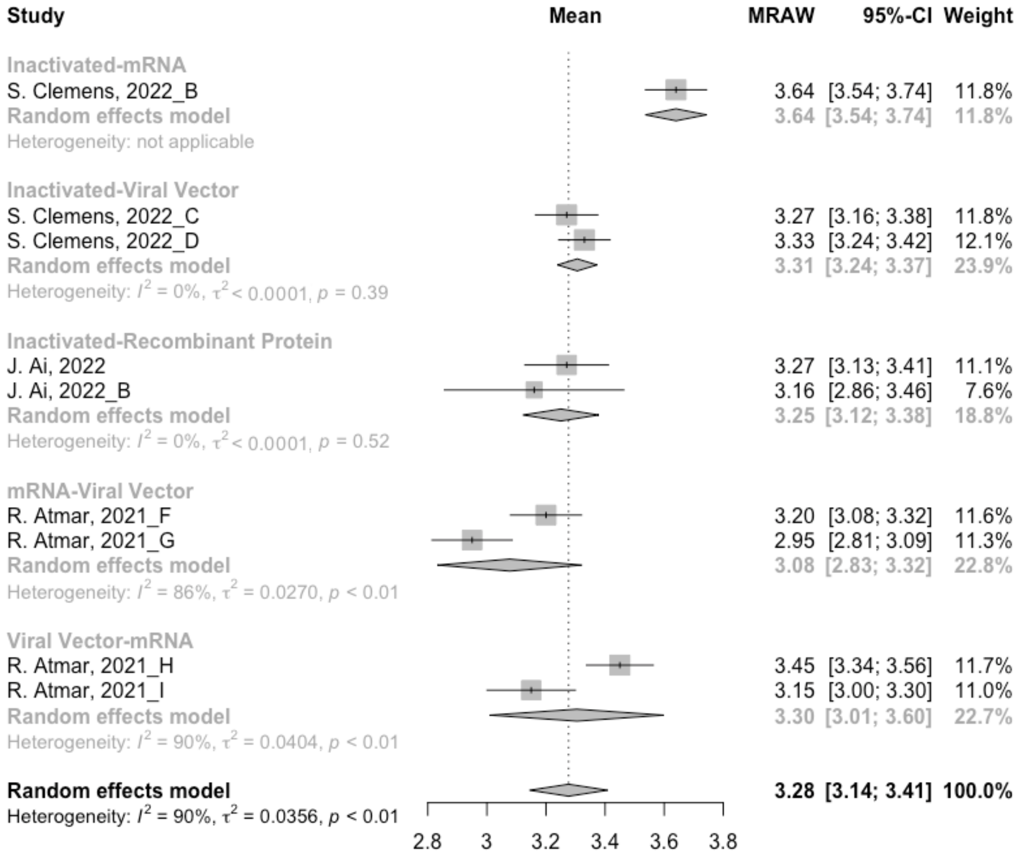

Figure S5. Forest plot of the pooled log-transformed neutralization titers against the wild type after heterogenous booster vaccination.

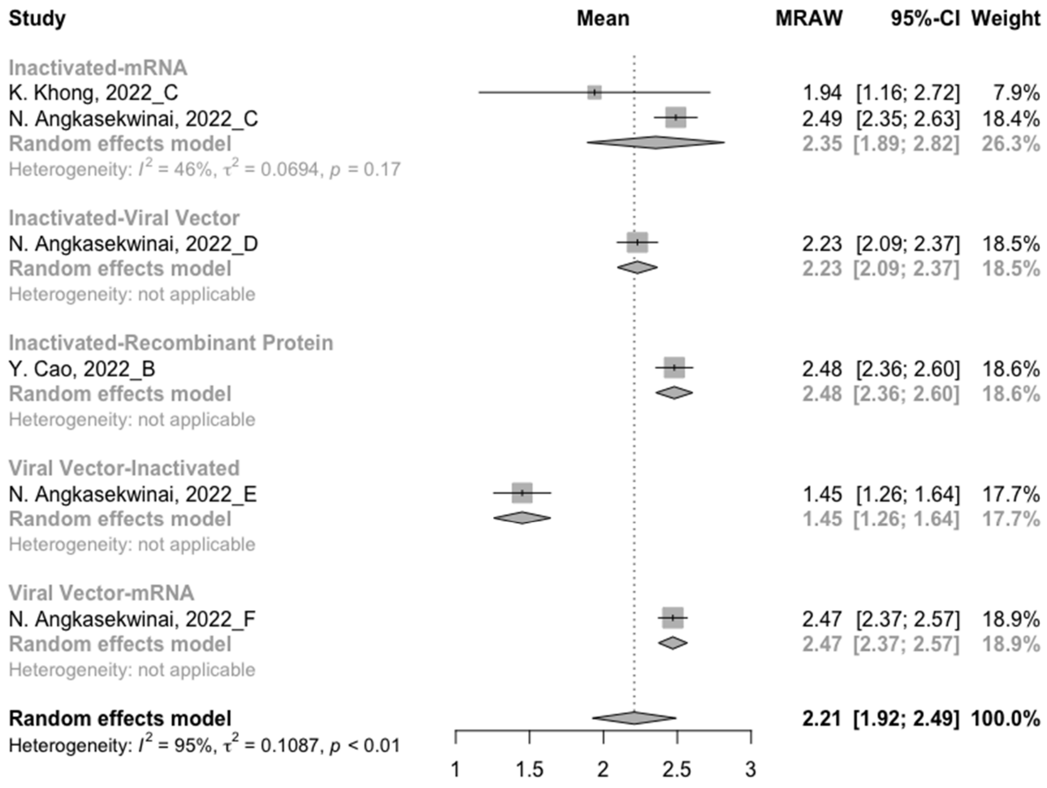

Figure S6. Forest plot of the pooled log-transformed neutralization titers against the Beta variant after heterogenous booster vaccination.

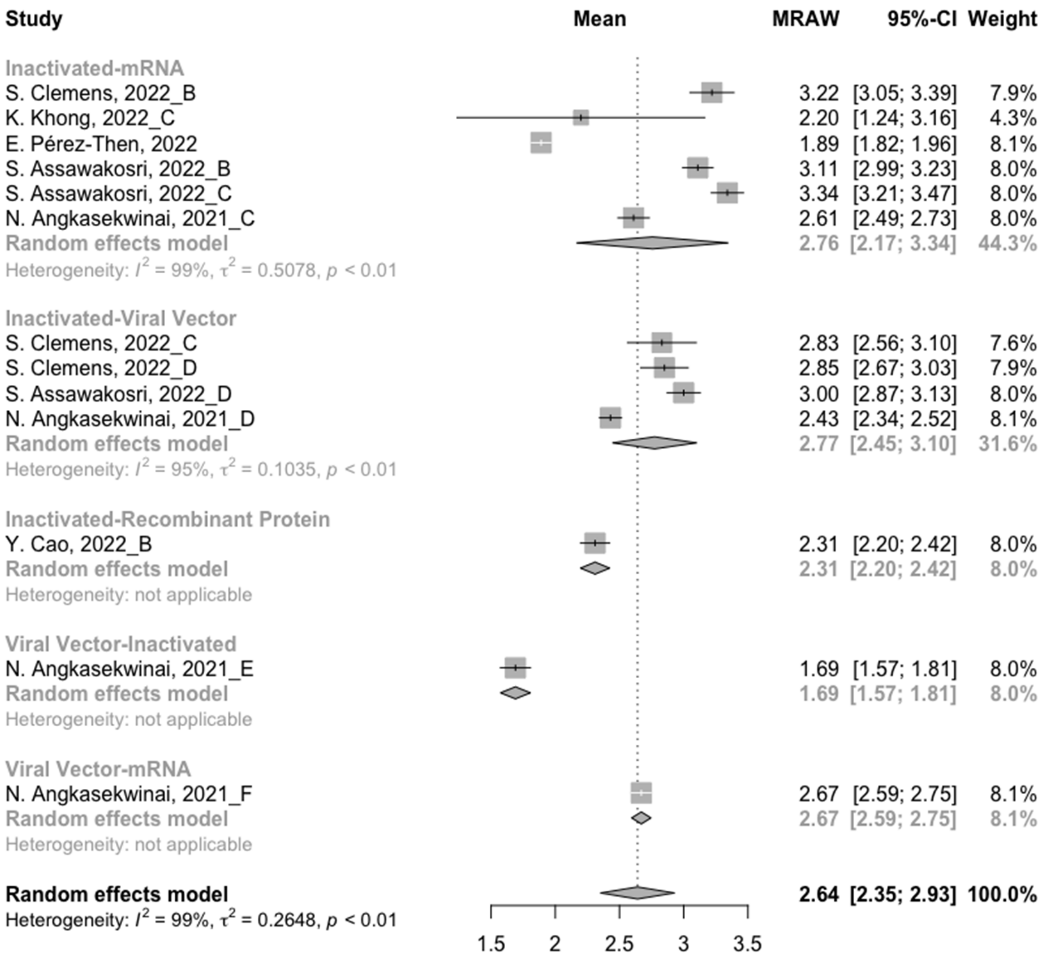

Figure S7. Forest plot of the pooled log-transformed neutralization titers against the Delta variant after heterogenous booster vaccination.

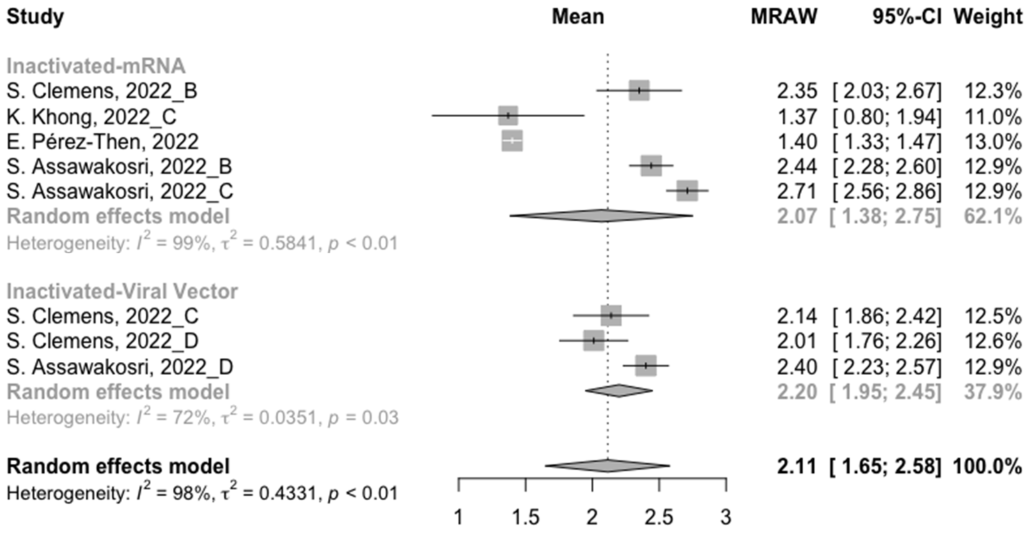

Figure S8. Forest plot of the pooled log-transformed neutralization titers against the Omicron variant after heterogenous booster vaccination.
